# During which period should we avoid cholecystectomy in patients who underwent endoscopic retrograde cholangiopancreatography?

**DOI:** 10.31744/einstein_journal/2020AO5393

**Published:** 2020-10-15

**Authors:** Murillo de Lima Favaro, Stefanie Bergamim Saviano Moran, Ana Paula Marconi Iamarino, Barbara Marrelli Herrero, Silvio Gabor, Marcelo Augusto Fontenelle Ribeiro

**Affiliations:** 1 Universidade de Santo Amaro São Paulo SP Brazil Universidade de Santo Amaro, São Paulo, SP, Brazil.; 2 Hospital do Servidor Público Estadual “Francisco Morato de Oliveira” São Paulo SP Brazil Hospital do Servidor Público Estadual “Francisco Morato de Oliveira”, São Paulo, SP, Brazil.

**Keywords:** Cholecystectomy, Cholelithiasis, Cholangiopancreatography, endoscopic retrograde, Laparoscopy

## Abstract

**Objective:**

To determine the period during which we should avoid cholecystectomy after endoscopic retrograde cholangiopancreatography.

**Methods:**

A retrospective analysis of electronic medical charts of 532 patients undergoing endoscopic retrograde cholangiopancreatography, between March 2013 and December 2017.

**Results:**

Approximately one-third of patients underwent the procedure between 4 and 30 days after endoscopic retrograde cholangiopancreatography. The conversion rate was 3.8%. The need for abdominal drainage and the finding of biliary tract injury after surgery were observed in 15.1% and 1.9% of patients, respectively. The length of stay was significantly shorter among patients undergoing surgery more than 30 days after endoscopic retrograde cholangiopancreatography. These patients had a median length of stay of one day, whereas the median length of stay in the group undergoing the procedure between 4 and 30 days after endoscopic retrograde cholangiopancreatography was 2 days.

**Conclusion:**

The period during which we should avoid cholecystectomy is between 4 and 30 days after endoscopic retrograde cholangiopancreatography.

## INTRODUCTION

Cholecystectomy is the treatment of choice for cholelithiasis and acute cholecystitis; however, in case of stones lodged in the common bile duct, cholecystectomy requires additional exploration of the biliary tract.^(^^1^^-^^3^^)^

Choledocholithiasis can be diagnosed with imaging methods, such as abdominal ultrasound, magnetic resonance (MR) cholangiography or intraoperative cholangiography, which identifies choledocholithiasis in approximately 10% of asymptomatic patients, which warrants its being used routinely.^(^^3^^-^^5^^)^

Endoscopic retrograde cholangiopancreatography (ERCP) is highly sensitive and specific for choledocholithiasis, but it is most frequently used after a confirmed diagnosis of choledocholithiasis, for therapeutic purposes.^(^^3^^,^^5^^-^^7^^)^

ERCP can be performed before, during or after cholecystectomy, and patients diagnosed with choledocholithiasis before surgical treatment or at high risk for complications, such as those with cholangitis or dilated biliary tree, must undergo preoperative ERCP. Lower-risk patients can undergo laparoscopic cholecystectomy with cholangiography and laparoscopic exploration of the common bile duct, depending on the surgeon's skills and the equipment available at the hospital.^(^^7^^-^^12^^)^

Usually, if choledocholithiasis is identified but not removed during cholecystectomy, a subsequent ERCP is required for extraction of the stones.^(^^13^^,^^14^^)^

Cholecystectomy must be performed safely, and inflammation resulting from the disease itself and manipulation during ERCP can hinder the surgery, increasing the operative time, the risk of bleeding and the conversion rate when compared with elective cholecystectomy without previous ERCP.^(^^13^^,^^15^^-^^19^^)^

Studies show that laparoscopic cholecystectomy (LC) and ERCP, performed more than 72 hours apart, lead to inflammation of the biliary tract, which may hinder the use of laparoscopy to approach the gallbladder and biliary ducts.^(^^20^^-^^26^^)^

In view of the need to prevent intra- and postoperative complications and their implications, so that a greater number of patients with an indication for LC after preoperative ERCP can be scheduled and treated the best way possible, at a hospital with a high demand for beds and limited financial resources, such as the *Hospital Geral do Grajaú* (HGG), a secondary care hospital in the city of São Paulo, it was imperative to assess which was the best period to perform LC in these patients.

## OBJECTIVE

To identify the period during which cholecystectomy should be avoided in patients previously undergoing endoscopic retrograde cholangiopancreatography.

## METHODS

A retrospective analysis of electronic medical records of 532 patients undergoing ERCP between March 2013 and December 2017, at HGG.

Informed consent forms were signed by all patients undergoing ERCP, and the study was approved by the Institutional Review Board of *Universidade de Santo Amaro* (UNISA), CAAE: 01867418.0.0000.0081, Approved Number # 3.018562.

Patients who waited at home for cholecystectomy were questioned over the phone about any complications or readmissions to the hospital.

**Figure 1:**
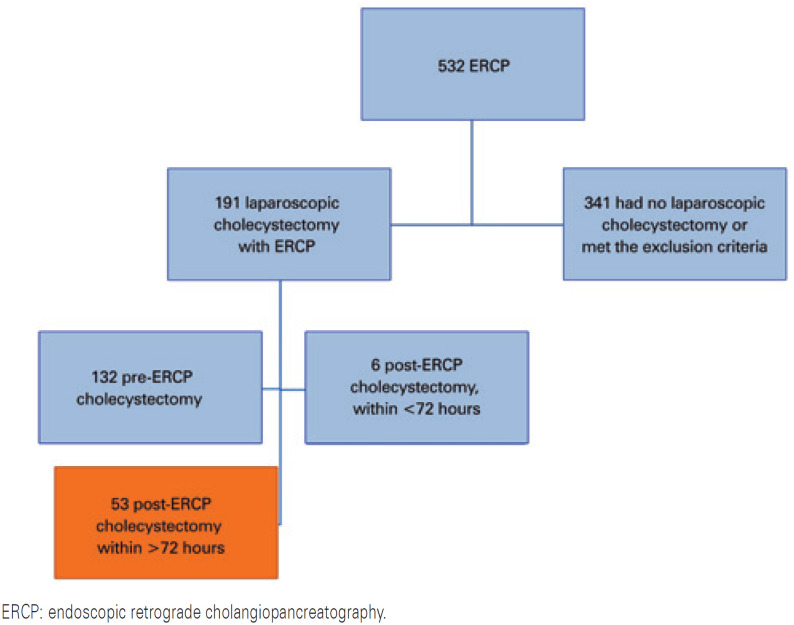
Selection of cases to be studied

The sample was defined by convenience sampling, including all consecutive patients with a prior diagnosis of choledocholithiasis, who were eligible throughout the duration of the study. We analyzed data (sex; age; time between ERCP and surgery; operative time; placement of surgical drain; conversion to open surgery; incidence of biliary injury; and hospital length of stay) from 53 patients. We excluded 341 cases in whom no cholecystectomy was performed after ERCP; open surgery was performed; or whose diagnosis or complications were cholangitis, cholecystitis, duodenal perforation, pancreatic or bile duct cancer, and also those undergoing cholecystectomy with biliary bypass. Patients subjected to laparoscopic cholecystectomy before ERCP were also excluded (Figure 1).

Patients enrolled in the study were divided into two groups based on the time between ERCP and surgery, as follows: 4 to 30 days, and more than 30 days.

Categorical variables were expressed as absolute values and relative frequencies, whereas continuous variables were described in measures of central tendency and dispersion. The Shapiro-Wilk test was used to verify the parametric nature of continuous numerical variables. The Mann-Whitney test was applied to assess age, operative time and length of stay for the two categories of time between ERCP and surgery. The association between demographic and clinical characteristics and other categorical outcomes, based on the ERCP-surgery time groups, was assessed by Pearson's χ^2^ test or Fisher's exact test, when indicated. Findings with a *p* value or probability of type I error <5% were considered statistically significant. The analyses were performed using Microsoft Excel 2010 and IBM (SPSS), version 21.0.

## RESULTS

The study sample was predominantly female (83.0%) with a median age of 47 years, ranging of 17-81 years. The median time between ERCP and the surgical procedure was 40 days. Slightly over one third of patients (41.5%) underwent the procedure within 4 to 30 days after ERCP. The median length of stay for the procedure was one day, and the median operative time was 150 minutes (Table 1).

**Table 1:** Characterization of the study sample

Characteristic		
Sex		
	Female	44 (83.0)
	Male	9 (17.0)
Age, years		
	Median (Minimum-Maximum)	47.0 (17-81)
	>60	8 (15.1)
ERCP-surgery time, days		
	Median (Minimum-Maximum)	40.0 (4-317)
	4-30	22 (41.5)
	>30	31 (58.5)
Length of stay (days)		
	Median (Minimum-Maximum)	1.0 (1-10)
Operative time (minutes)*		
	Median (Minimum-Maximum)	150.0 (80-260)
Frequency of outcomes		
	Conversion†	2 (3.8)
	Abdominal drain	8 (15.1)
	Biliary duct injury	1 (1.9)

Except when stated otherwise, results are expressed in n (%).

* Missing values n=3 (5.6%);

† Missing values n=1 (1.9%).

ERCP: endoscopic retrograde cholangiopancreatography.

The conversion rate of the procedure was 3.8%. The need for abdominal drainage and occurrence of biliary injury after the surgery were 15.1% and 1.9%, respectively.

Table 2 shows the characteristics of the sample for the different ERCP-surgery times. There were no differences in general characteristics between the two groups. Women were more prevalent in both groups, and the median age of patients in the group who underwent the procedure within 4 to 30 days after ERCP, was slightly higher than that of the group within more than 30 days.

**Table 2:** Characterization of the study sample for the different times between endoscopic retrograde cholangiopancreatography and surgery

Characteristics		4-30 days (n=22)	>30 days (n=31)	p value
Sex				
	Female	19 (86.4)	25 (80.6)	0.585
	Male	3 (13.6)	6 (19.4)	
Age, years				
	Median (Minimum-Maximum)	51.0 (17-81)	42.0 (19-80)	0.338
	≥60	5 (22.7)	3 (9.7)	0.253*
ERCP-surgery time, days				
	Median (Minimum-Maximum)	10.5 (4-29)	62.0 (33-317)	

Except when stated otherwise, results are expressed in n (%).

* Fisher's exact test.

ERCP: endoscopic retrograde cholangiopancreatography.

**Figure 2:**
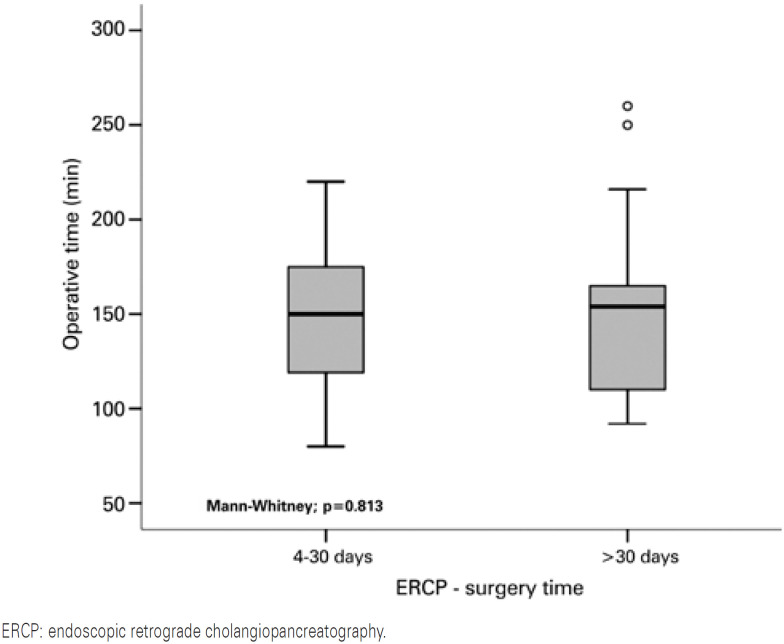
Operative time as per time between endoscopic retrograde cholangiopancreatography and surgery

We did not find any significant differences in the operative time distribution between the two ERCP-surgery time groups (p=0.813). The median procedure time was 150 minutes (80-220) in the group undergoing the procedure within 4 to 30 days, and 154.0 (92-260) minutes for the group within more than 30 days (Figure 2).

**Figure 3:**
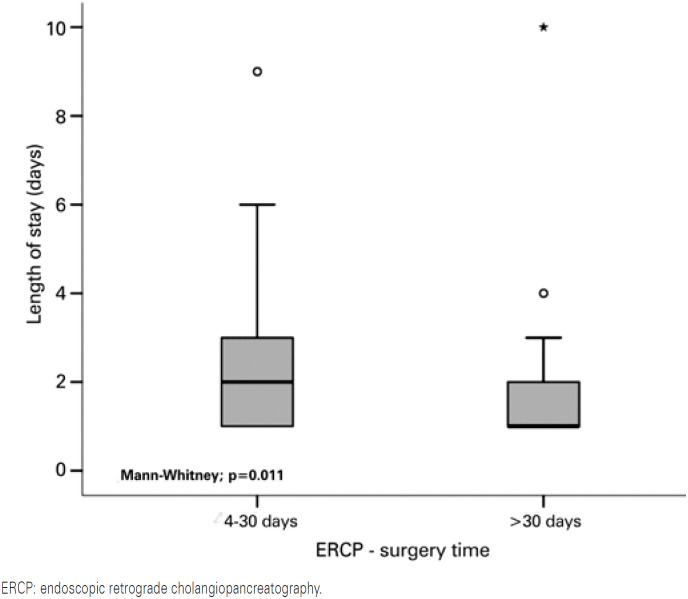
Length of stay as per time between endoscopic retrograde cholangiopancreatography and surgery

The hospital length of stay was significantly shorter in the group with ERCP-surgery time of more than 30 days (p=0.011). These patients had a median length of stay of 1 (1-10) day. The median length of stay in the group undergoing the procedure within 4 to 30 days was 2 (1-9) days, as shown in figure 3.

**Figure 4:**
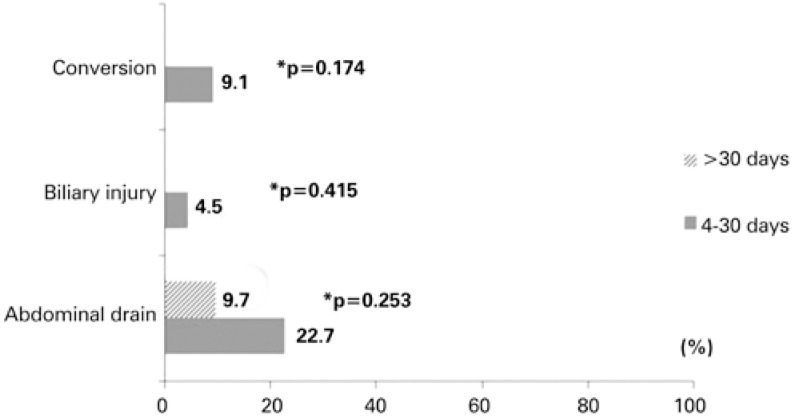
Frequency of outcomes as per time between endoscopic retrograde cholangiopancreatography and surgery

Figure 4 shows the frequency of the outcomes - conversion rate, biliary injury, and need for abdominal drainage, as per time between ERCP and surgery. Biliary injury, conversion rate and need for abdominal drainage were more frequent in the group undergoing the procedure within 4 to 30 days.

Using telephone contact or outpatient evaluation, we successfully identified non-readmission and absence of pain symptoms or relapse during the waiting interval for LC.

## DISCUSSION

In line with the literature, demographic differences (sex or age) had no statistical relevance in this study, and women were more prevalent among all patients investigated.^(^^21^^)^

The mean operative time of elective LC found in the studies ranged from 46 to 76 minutes, depending on the skills of the surgical team.^(^^27^^)^ At HGG, elective laparoscopic cholecystectomies had a mean operative time of 148.7 minutes. This difference can be explained by the learning curve of general surgery residents. There was no difference in the mean operative time between the two different times between ERCP and surgery.

The mean postoperative length of stay was greater among patients who underwent laparoscopic cholecystectomy 4 to 30 days after ERCP, whereas those who underwent LC more than 30 days after ERCP had the shortest postoperative length of stay. Patients who underwent LC within 3 days from ERCP had a shorter length of stay than those whose surgery was performed more than 3 days after ERCP. Nonetheless, we did not investigate which was length of stay was shorter between the later intervals.^(^^28^^)^ In our study, we showed that patients operated in the latest interval (more than 30 days after ERCP) had a shorter median postoperative length of stay of 1 day against 2 days, with statistical significance.

Despite the inflammatory complications of ERCP, as described by Daher Filho et al.,^(^^29^^)^ this study showed that most LC are finished by videolaparoscopy, and only 4.87% required conversion to open surgery. The LC requiring conversion to open surgery were performed 4 to 30 days after ERCP, with a conversion rate of 9.1%, which supports the data presented.^(^^18^^,^^19^^,^^28^^,^^30^^)^

Although a very individual choice of each surgeon, abdominal cavity drainage in cholecystectomies, in our service, is only used in surgical procedures leading to technical difficulties and when the surgical team is unsure about potential iatrogenic injury or bleeding in the liver bed. According to Fortunato et al., LC performed immediately after or up to 3 days after ERCP reduces the risk of injury during the surgical procedure and the need for abdominal cavity drainage.^(^^30^^)^ Between 4 to 30 days after ERCP, 22.7% of patients required abdominal cavity drainage, and 4.5% suffered biliary injury. However, patients who underwent LC more than 30 days after ERCP did not have biliary injury, and only 9.7% required an abdominal cavity drain, proving that the inflammatory process initiated 72 hours after ERCP leads to more complexity for performance of LC,^(^^27^^)^ and after 30 days from the ERCP, the complication rate is lower.

Cholecystectomy early after ERCP, within 72 hours, has the best outcomes with less morbidity, probably due to the fact that the inflammatory process triggered by ERCP is not yet established.^(^^13^^)^ However, at HGG, like in most secondary care hospitals of the public health system, most LC are conducted more than 3 days after ERCP. Thus, when looking at later periods, the best time to perform LC in patients with previous ERCP is preferably 30 days or more after the first procedure, due to better outcomes in terms of operative time, biliary injury rate, need for abdominal cavity drainage, conversion rate and postoperative length of stay. The worst period, when looking at the same variables, is 4 to 30 days after ERCP.^(^^9^^,^^10^^)^

## CONCLUSION

In this study sample, the period during which we should avoid performing cholecystectomy after endoscopic retrograde cholangiopancreatography is within 4 to 30 days.
