# Supplementary material for: During which period should we avoid cholecystectomy in patients who underwent endoscopic retrograde cholangiopancreatography?
Source: Einstein (Sao Paulo). 2020 Oct 15;18:eAO5393. doi: 10.31744/einstein_journal/2020AO5393 (PMC9586429; doi:10.31744/einstein_journal/2020AO5393)
Supplement: Translation

# Translation: Durante qual período devemos evitar a colecistectomia em pacientes que realizaram colangiopancreatografia retrógrada endoscópica?

**Authors:** Murillo de Lima Favaro, Stefanie Bergamim Saviano Moran, Ana Paula Marconi Iamarino, Barbara Marrelli Herrero, Silvio Gabor, Marcelo Augusto Fontenelle Ribeiro

**Affiliations:** 1 São Paulo SP Brasil Universidade de Santo Amaro, São Paulo, SP, Brasil.; 2 São Paulo SP Brasil Hospital do Servidor Público Estadual “Francisco Morato de Oliveira”, São Paulo, SP, Brasil.

**Keywords:** Colecistectomia, Colelitíase, Colangiopancreatografia retrógrada endoscópica, Laparoscopia

## Abstract

**Objetivo:**

Analisar o período durante o qual devemos evitar a colecistectomia após a colangiopancreatografia retrógrada endoscópica.

**Métodos:**

Foi realizada análise retrospectiva dos prontuários eletrônicos de 532 pacientes submetidos à colangiopancreatografia retrógrada endoscópica, no período de março de 2013 a dezembro de 2017.

**Resultados:**

Cerca de um terço dos pacientes realizaram o procedimento entre 4 e 30 dias após a colangiopancreatografia retrógrada endoscópica. A frequência de conversão do procedimento foi de 3,8%. A necessidade de dreno abdominal e o achado de lesão de via biliar após a cirurgia atingiram 15,1% e 1,9% dos pacientes, respectivamente. O tempo de internação foi significativamente menor entre os pacientes com tempo de colangiopancreatografia retrógrada endoscópica superior a 30 dias. Estes pacientes apresentaram, em mediana, um dia de internação hospitalar, enquanto o tempo mediano de internação no grupo que realizou o procedimento entre 4 e 30 dias foi de 2 dias.

**Conclusão:**

O período durante o qual devemos evitar realizar a colecistectomia após a colangiopancreatografia retrógrada endoscópica é do 4° ao 30° dia.

## INTRODUÇÃO

A colecistectomia é o tratamento de escolha da colelitíase e da colecistite aguda, mas, quando são identificados cálculos em via biliar principal, a colecistectomia necessita de complementação, com exploração das vias biliares.^(^^1^^-^^3^^)^

O diagnóstico da coledocolitíase pode ser feito a partir de métodos de imagem, como o ultrassom abdominal, a colangiorressonância ou a colangiografia intraoperatória, a qual identifica coledocolitíase em aproximadamente 10% de pacientes assintomáticos, sendo um importante argumento para ser realizada rotineiramente.^(^^3^^-^^5^^)^

A colangiopancreatografia retrógrada endoscópica (CPRE) é altamente sensível e específica para coledocolitíase, mas sua maior utilização se dá após diagnóstico confirmado de coledocolitíase, tendo função terapêutica.^(^^3^^,^^5^^-^^7^^)^

A CPRE pode ser realizada antes, durante ou após a colecistectomia, sendo que, os pacientes em que a coledocolitíase é diagnosticada antes do tratamento cirúrgico ou com risco elevado de complicação, como aqueles com colangite ou árvore biliar dilatada, devem ser submetidos à CPRE pré-operatória. Aqueles que têm risco mais baixo podem ser submetidos à colecistectomia laparoscópica com colangiografia e exploração laparoscópica do colédoco, dependendo da habilidade do cirurgião e do material disponível em seu hospital.^(^^7^^-^^12^^)^

Geralmente, uma coledocolitíase identificada, mas não removida, durante a colecistectomia, exige CPRE posterior para a retirada do cálculo.^(^^13^^,^^14^^)^

A colecistectomia deve ser executada de forma segura, mas um quadro de inflamação decorrente da própria doença e da manipulação pela CPRE pode dificultar o ato cirúrgico, com aumento do tempo operatório, maior risco de sangramento e taxas de conversão maiores do que quando a colecistectomia é realizada de forma eletiva sem CPRE prévia.^(^^13^^,^^15^^-^^19^^)^

Estudos mostram que a colecistectomia videolaparoscópica (CCC VLP) e a CPRE, realizadas com um intervalo maior que 72 horas, apresentam alterações inflamatórias nas vias biliares, que podem dificultar a abordagem da vesícula e vias biliares por videolaparoscopia.^(^^20^^-^^26^^)^

Perante a necessidade de se evitarem complicações intra e pós-operatórias e suas implicações, para que um número maior de pacientes que tenham indicação para CCC VLP, após serem submetidos à CPRE pré-operatória, possam ser agendados e tratados da melhor forma possível em um hospital com alta demanda de leitos e recursos financeiros limitados, como o Hospital Geral do Grajaú (HGG), um hospital secundário de São Paulo, fez-se necessário analisar qual o melhor período para realizar a CCC VLP nesses pacientes.

## OBJETIVO

Identificar o período durante o qual deve-se evitar a realização da colecistectomia em pacientes submetidos previamente à colangiopancreatografia retrógrada endoscópica.

## MÉTODOS

Foi realizada uma análise retrospectiva dos prontuários eletrônicos de 532 pacientes submetidos à CPRE, no período de março de 2013 a dezembro de 2017 no HGG.

Os Termos de Consentimento foram assinados por todos pacientes submetidos à CPRE, e o trabalho foi submetido ao Comitê de Ética da Universidade de Santo Amaro (UNISA), CAAE: 01867418.0.0000.0081, obtendo aprovação, conforme número do parecer 3.018562.

Os pacientes que aguardaram em casa pela realização da colecistectomia foram questionados em relação a complicações ou reinternações por meio de contato telefônico.

A definição da amostra se deu pelo critério de conveniência, que incluiu, consecutivamente, todos os pacientes com diagnóstico prévio de coledocolitíase elegíveis durante o período de estudo. Foram analisados os dados (sexo; idade; tempo entre a CPRE e a cirurgia; tempo cirúrgico; introdução de dreno cirúrgico; conversão para cirurgia aberta; ocorrência de lesão de via biliar; e tempo de permanência hospitalar) de 53 pacientes. Foram excluídos 341 casos em que não foi realizada colecistectomia após a CPRE; foi realizada a técnica aberta; ou os que tiveram como diagnóstico ou complicação colangite, colecistite, perfuração duodenal, neoplasia de pâncreas ou de vias biliares, e também os que realizaram derivação de vias biliares associada à colecistectomia. Foram também excluídos os que realizaram a CCC VLP antes da CPRE (Figura 1).

**Figura 1:** Seleção dos casos a serem estudados

Os pacientes incluídos no estudo foram divididos em dois grupos baseados no período que a cirurgia foi realizada após a CPRE, a saber: 4 a 30 dias e mais de 30 dias.

As variáveis categóricas foram apresentadas em valores absolutos e frequências relativas, enquanto as contínuas foram descritas em medidas de tendência central e dispersão.

O teste de Shapiro-Wilk foi utilizado para verificação da natureza paramétrica das variáveis numéricas contínuas. O teste de Mann-Whitney foi aplicado para a avaliação da idade e dos tempos de procedimento cirúrgico e internação hospitalar entre as categorias de tempo CPRE-cirurgia. A associação entre características demográficas e clínicas e outros desfechos categóricos, segundo os grupos de tempo CPRE-cirurgia, foi avaliada por meio do teste do χ^2^ de Pearson ou do teste exato de Fischer, quando indicado. Foram considerados achados estatisticamente significativos aqueles com valor de p ou probabilidade de erro tipo I <5%. As análises foram realizadas com auxílio dos programas Microsoft Excel 2010 e IBM (SPSS), versão 21.0.

## RESULTADOS

A amostra estudada foi predominantemente feminina (83,0%) com idade mediana de 47 anos, variando entre 17 e 81 anos completos. O tempo mediano entre a realização da CPRE e o procedimento cirúrgico foi de 40 dias. Pouco mais de um terço dos pacientes (41,5%) realizou o procedimento entre 4 e 30 dias após a CPRE. O tempo de internação hospitalar para a realização do procedimento foi, em mediana, um dia, e o tempo mediano de procedimento foi 150 minutos (Tabela 1).

**Tabela 1:** Caracterização da amostra estudada

Característica		
Sexo		
	Feminino	44 (83,0)
	Masculino	9 (17,0)
Idade, anos		
	Mediana (Mínimo-Máximo)	47,0 (17-81)
	>60	8 (15,1)
Tempo CPRE-cirurgia, dias		
	Mediana (Mínimo-Máximo)	40,0 (4-317)
	4-30	22 (41,5)
	>30	31 (58,5)
Tempo de internação (dias)		
	Mediana (Mínimo-Máximo)	1,0 (1-10)
Tempo de cirurgia (minutos)*		
	Mediana (Mínimo-Máximo)	150,0 (80-260)
Frequência de desfechos		
	Conversão†	2 (3,8)
	Dreno abdominal	8 (15,1)
	Lesão via biliar	1 (1,9)

Quando não indicado de outra forma, resultados expressos em n (%).

* Valores ausentes n=3 (5,6%);

† Valores ausentes n=1 (1,9%).

CPRE: colangiopancreatografia retrógrada endoscópica.

A frequência de conversão do procedimento foi de 3,8%. A necessidade de dreno abdominal e o achado de lesão de via biliar após a cirurgia foram observados em 15,1% e 1,9% dos pacientes, respectivamente.

A tabela 2 apresenta as características da amostra, segundo períodos de tempo, entre a realização da CPRE e o procedimento cirúrgico. Não foi possível observar diferenças nas características gerais entre os grupos. A frequência de mulheres foi maior em ambos os grupos de tempo, e a idade mediana dos pacientes do grupo que realizou o procedimento entre 4 e 30 dias foi ligeiramente superior à observada no grupo que o fez com mais de 30 dias.

**Tabela 2:** Caracterização da amostra estudada, segundo períodos de tempo entre a colangiopancreatografia retrógrada endoscópica e a cirurgia

Características		4-30 dias (n=22)	>30 dias (n=31)	Valor de p
Sexo				
	Feminino	19 (86,4)	25 (80,6)	0,585
	Masculino	3 (13,6)	6 (19,4)	
Idade, anos				
	Mediana (Mínimo-Máximo)	51,0 (17-81)	42,0 (19-80)	0,338
	≥60	5 (22,7)	3 (9,7)	0,253*
Tempo CPRE-cirurgia, dias				
	Mediana (Mínimo-Máximo)	10,5 (4-29)	62,0 (33-317)	

Quando não indicado de outra forma, resultados expressos em n (%).

* teste exato de Fisher.

CPRE: colangiopancreatografia retrógrada endoscópica.

Não foi possível observar diferenças significativas na distribuição do tempo de procedimento segundo os grupos de tempo CPRE-cirurgia (p=0,813). O tempo mediano de duração do procedimento foi de 150 (80-220) minutos no grupo que o realizou entre 4 e 30 dias e de 154,0 (92-260) minutos para o grupo com tempo superior a 30 dias (Figura 2).

**Figura 2:** Tempo de duração do procedimento em relação ao tempo colangiopancreatografia retrógrada endoscópica cirurgia

O tempo de internação foi significativamente menor entre os pacientes do grupo de tempo CPRE-cirurgia superior a 30 dias (p=0,011). Estes pacientes apresentaram, em mediana, 1 (1 a 10) dia de internação hospitalar. Já o tempo mediano de internação no grupo que realizou o procedimento entre 4 e 30 dias foi de 2 (1 a 9) dias, conforme ilustrado na figura 3.

**Figura 3:** Tempo de internação em relação ao tempo colangiopancreatografia retrógrada endoscópica cirurgia

A figura 4 apresenta a frequência dos desfechos - conversão do procedimento, lesão de via biliar e necessidade de dreno abdominal, segundo os períodos de tempo entre CPRE e cirurgia. A frequência de lesão biliar, conversão e necessidade de dreno abdominal foi maior no grupo que executou o procedimento entre 4 e 30 dias.

**Figura 4:** Frequência de desfechos em relação ao tempo de realização de colecistectomia após colangiopancreatografia retrógrada endoscópica cirurgia

Por meio de contato telefônico ou avaliação ambulatorial, constatamos que houve sucesso no que se referiu a não reinternação e à ausência de sintomas de dor ou recorrência do quadro durante o intervalo de espera para a realização da CCC VLP.

## DISCUSSÃO

Assim como evidenciado na literatura, as diferenças demográficas (sexo ou idade) não tiveram relevância estatística no presente estudo, sendo as mulheres mais prevalentes entre todos os pacientes analisados.^(^^21^^)^

O tempo médio de CCC VLP eletiva encontrado nos estudos analisados variou desde 46 até 76 minutos, a depender da experiência da equipe cirúrgica.^(^^27^^)^ No HGG, as colecistectomias VLP eletivas apresentaram tempo médio de 148,7 minutos. Essa diferença pode ser explicada pela curva de aprendizado dos residentes da cirurgia geral. Não foi encontrada diferença na média dos tempos cirúrgicos entre os intervalos entre a CPRE e a cirurgia.

O tempo médio de internação pós-operatória foi maior entre os pacientes que realizaram colecistectomia VLP no intervalo entre 4 e 30 dias após a CPRE, enquanto os submetidos à CCC VLP depois do 30° dia da CPRE tiveram o menor tempo de internação pós-operatória. Pacientes que realizaram CCC VLP até 3 dias após CPRE têm tempo de internação menor do que aqueles que realizaram a cirurgia após o terceiro dia da CPRE, porém não foi analisado qual o menor tempo de internação nos intervalos tardios.^(^^28^^)^ No nosso estudo, evidenciamos que pacientes operados no intervalo tardio (depois do 30° dia da CPRE) têm menor hospitalização pós-operatória mediana de 1 dia contra 2 dias, apresentando significância estatística.

Apesar das complicações inflamatórias geradas pela CPRE, descritas por Daher Filho et al.,^(^^29^^)^ o presente estudo mostrou que a maioria das CCC são finalizadas por videolaparoscopia, e apenas 4,87% destas necessitaram serem convertidas à CCC aberta. Essas CCC que necessitaram conversão para via aberta foram realizadas no intervalo do 4° ao 30° dia após a CPRE, com taxa de conversão de 9,1% das cirurgias realizadas nesse intervalo, corroborando dados evidenciados.^(^^18^^,^^19^^,^^28^^,^^30^^)^

Apesar de ser uma escolha muito particular de cada cirurgião, a necessidade de drenagem da cavidade abdominal nas colecistectomias, em nosso serviço, somente é utilizada nos procedimentos cirúrgicos que tenham gerado dificuldades técnicas e dúvida para equipe cirúrgica quanto a possíveis lesões iatrogênicas ou sangramento em leito hepático. Conforme Fortunato et al., a CCC VLP realizada logo após a CPRE ou em até 3 dias diminui o risco em causar lesões durante o procedimento cirúrgico e a necessidade de drenagem da cavidade abdominal.^(^^30^^)^ No intervalo de 4 a 30 dias após a CPRE, 22,7% dos pacientes necessitaram utilização de dreno na cavidade abdominal, e 4,5% sofreram lesão em via biliar. Porém, os pacientes submetidos à CCC VLP após o 30º dia da CPRE não sofreram lesão em via biliar, e somente 9,7% necessitaram colocação de dreno em cavidade abdominal, comprovando que o processo inflamatório iniciado 72 horas após a CPRE gera maior complexidade para realização da CCC VLP,^(^^27^^)^ e, após o 30º da CPRE, há menos complicações operatórias.

A colecistectomia precoce após CPRE, dentro de 72 horas, tem melhores resultados com menos morbidades, provavelmente por ainda não ter se instalado o processo inflamatório gerado após a CPRE.^(^^13^^)^ Porém, no HGG, como em grande parte dos hospitais secundários do sistema público de saúde, a maioria das CCC VLP é realizada a partir do 3° dia após a CPRE. Logo, ao analisar os períodos tardios, o melhor momento para realizar CCC VLP nos pacientes submetidos à CPRE é preferencialmente depois do 30° dia após o procedimento, por conta de menores duração cirúrgica, taxa de lesão de vias biliares, necessidade em drenagem de cavidade abdominal, taxa de conversão da via de acesso e menor tempo de internação pós-operatório. O pior momento, quando analisadas essas mesmas variáveis, é no intervalo entre o 4° e 30° dia após a CPRE.^(^^9^^,^^10^^)^

## CONCLUSÃO

Na amostra estudada, o período durante o qual se deve evitar a realização de colecistectomia em pacientes que submetidos à colangiopancreatografia retrógrada endoscópica foi o intervalo entre o 4° o 30° dia.
